# Optimal treatment determination on the basis of haematoma volume and intra-cerebral haemorrhage score in patients with hypertensive putaminal haemorrhages: a retrospective analysis of 310 patients

**DOI:** 10.1186/1471-2377-14-141

**Published:** 2014-07-04

**Authors:** Hao Liu, Yunhui Zen, Jin Li, Xiang Wang, Hao Li, Jianguo Xu, Chao You

**Affiliations:** 1 Department of Neurosurgery, West China Hospital, Sichuan University, 37 Guoxue Street, 610041 Chengdu, Sichuan, People's Republic of China

**Keywords:** Spontaneous putaminal haemorrhage, Intra-cerebral haemorrhage score, Surgical indication, Intra-cerebral haemorrhage

## Abstract

**Background:**

Hypertensive putaminal haemorrhage comprises major part of intra-cerebral haemorrhages, with particularly high morbidity and mortality. However, the optimal treatments for these individuals remain controversial.

**Methods:**

From June 2010 to August 2013, patients with hypertensive putaminal haemorrhages were treated in the Department of Neurosurgery, West China Hospital. Information regarding the age, signs of cerebral herniation, haematoma volume, intra-ventricular haemorrhage, intra-cerebral haemorrhage score and the treatments of each patient were analyzed retrospectively. The outcome was evaluated by the 30-day mortality rate.

**Results:**

The 30-day mortality rate of the patients with haematomas volume greater than or equal to 30 ml and intra-cerebral haemorrhage scores of 1 or 2 was decreased in the surgical group compared with those in the conservative group (1.92% VS. 21.40%, OR = 0.072, p = 0.028; 15.40% VS. 33.3%, OR = 0.365, p = 0.248, respectively). The mortality rate of the patients with signs of cerebral herniation was not significantly different between the surgical and conservative groups (83.30% VS. 100%; p = 0.529). The intra-cerebral haemorrhage score was significantly associated with the 30-day mortality rate of patients with intra-cerebral haemorrhages (r = -0.798, p < 0.001).

**Conclusion:**

Patients with basal ganglia haematomas volume greater than or equal to 30 ml and intra-cerebral haemorrhage scores of 1 or 2 could benefit from the surgical removal of haematomas. The intra-cerebral haemorrhage score can accurately predict the 30-day mortality rate of patients with hypertensive putaminal haemorrhages.

## Background

A spontaneous intra-cerebral haemorrhage (ICH) is a devastating subtype of stroke with an annual incidence of 20–30 per 1,000,000 people [1]. Haematomas within the basal ganglia comprise 60% of all cases with hypertensive ICHs, and these cases have a particularly high morbidity and mortality despite optimized treatments [2]. Dennis MS et al. have reported that the one-year survival rate of patients with these haematomas is only 38% [3] and that most survivors are disabled [4]. However, the role of surgical and medical therapy in treating putaminal haemorrhage remains controversial. Although the Surgical Trial in Intracerebral haemorrhage (STICH) has shown that there is no significant benefit of early surgery compared with initial conservative treatment in patients with spontaneous ICHs [5], trials in Japan have shown that early surgery can improve the mortality of patients with spontaneous ICHs [6]. Furthermore, several recent studies have suggested that haematoma clot reduction can limit the brain oedema and local ischaemia, as well as the severity of the neurological deficits that are observed after an ICH [7]. Hence, we believed that a subset of patients with basal ganglia haemorrhages could benefit from the micro-surgical evacuation of haematomas. The ICH score is a simple and reliable clinical grading scale that is used for predicting the early mortality of patients with ICHs [8]. However, there are no existing data regarding treatment selection on the basis of haematoma volume and the ICH score of patients with basal ganglia haemorrhages. Therefore, we conducted a retrospective study to investigate whether the patients with hypertensive putaminal haemorrhages who can benefit from the surgical removal of haematomas on the basis of the haematoma volume and ICH score.

## Methods

From June 2010 to August 2013, the medical records of patients with hypertensive basal ganglia haemorrhages who were admitted to the Department of Neurosurgery of West China Hospital were retrospectively reviewed. Information on their basic clinical characteristics, treatments and 30-day outcomes after the ictus was collected. The exclusion criteria were as follows: 1) patients with haemorrhages caused by cerebral aneurysms, arteriovenous malformations, brain injuries, cerebral tumours or infarctions, and patients who did not undergo computed tomography cerebral angiography; 2) patients with dysfunctions of coagulation, liver cirrhosis, uremia, heart failure, chronic obstructive pulmonary disease or pregnancy; 3) patients on anticoagulant therapy; 4) patients with haemorrhagic conversion after stoke.

**Figure 1:**
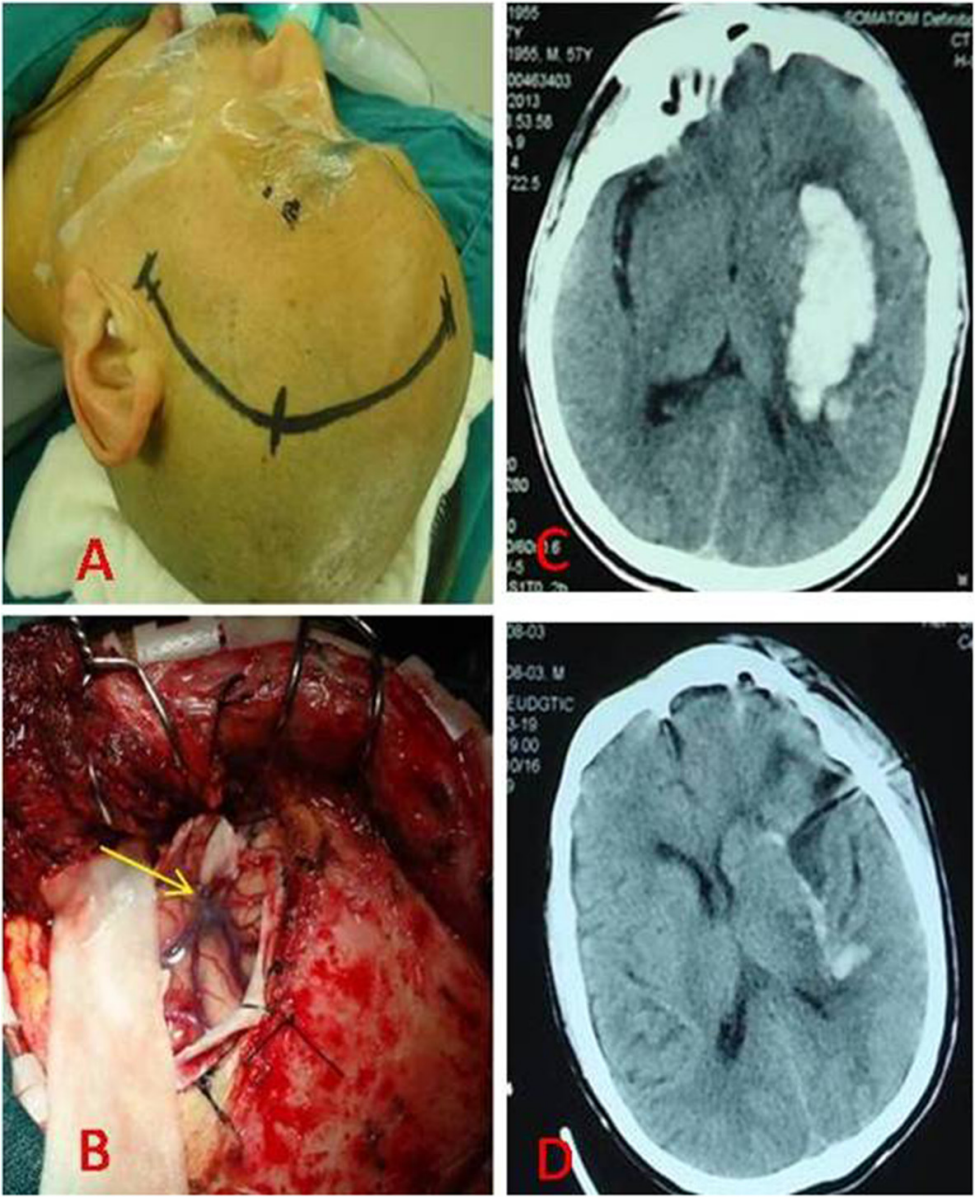
**The basal ganglia hematoma was evacuated by transsylvian-transinsular approach. A**: A small question mark incision line for the frontal-temporal craniotomy; **B**: The dural was opened, the sylvian was seen (yellow arrow); **C**: The CT scan demonstrated a large hematoma within the left basal ganglia of the head, when the patient was admission to neurosurgery department; **D**: postoperative CT scan showed the intracranial hematoma was totally removed and there was no obvious parenchymal injuries.

**Figure 2:**
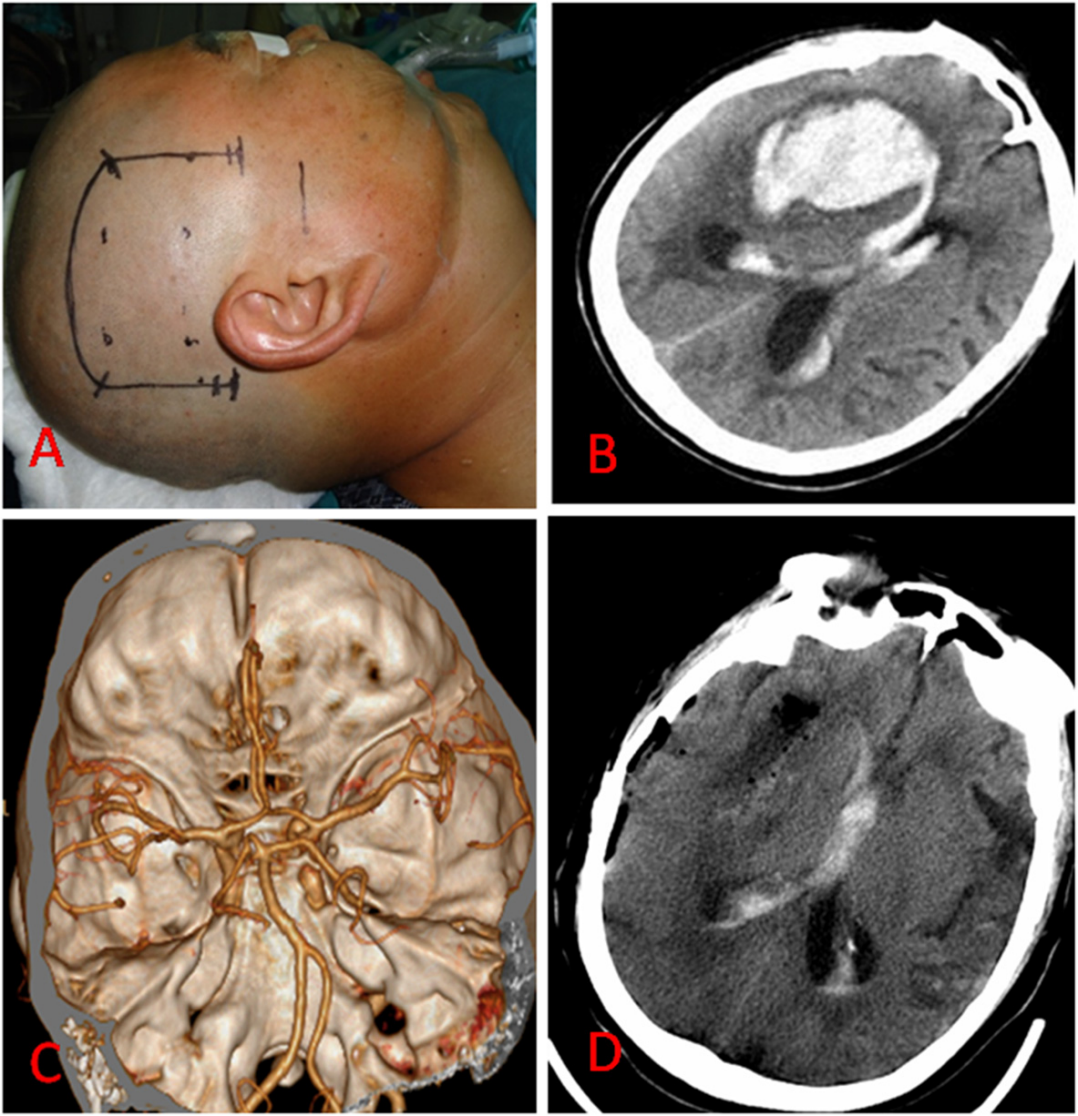
**The basal ganglia hematoma was evacuated by Transcortical-transtemporal approach. A**: A horseshoe shape incision line for the parietal-temporal craniotomy; **B**: The CT scan demonstrated a large hematoma within the right basal ganglia of the head and intraventricular hemorrhage, when the patient was admission to neurosurgery department; **C**: both of aneurysm and AVM were found on CTA; **D**: CT scan showed the hematoma within the basal ganglia except of the intriventriclar hemorrhage, was totally removed on postoperative day 2.

All of the variables that were possibly associated with the outcomes of patients with hypertensive basal ganglia hemorraghe were evaluated. Age, signs of cerebral herniation (dilated and fixed pupils), haematoma volume, intra-ventricular haemorrhage, surgical intervention and ICH score were recorded. The haematoma volume was calculated with the planimetric method (ABC/2) [9]. Furthermore, the presence of an intra-ventricular haemorrhage was noted at the initial computed tomographic scan. The patients received surgical haematoma removal through the transsylvian-transinsular approach (Figure 1) or the transcortical-transtemporal approach (Figure 2), and all of the operations were performed within 72 h of the ictus.

The surgical team included three skilled professors, and the surgical procedure was determined by the neurosurgeon. If the patients with basal ganglia hematoma volume ≥ 30 ml, shift of the midline ≥ 5 mm, compression of the ventricle ≥ 20%, or signs of cerebral herniation, the surgical intervention was mandatory according to the protocol of neurosurgery department. Mortality was assessed 30 days after the ICH ictus. Comatose patients who were discharged voluntarily by their relatives were recorded as deaths. Conscious patients who were discharged were interviewed over telephone 30 days after the ictus. The patients or their relatives signed informed consents after a thorough explanation of the surgical or conservative treatments. The patients signed the informed consent for the publication of their clinical images. The trial was approved by the Biological and Medical Ethics Committee of West China Hospital.

### Statistical analysis

The statistical analysis was performed using SPSS 19.0 software (IBM Corporation, Armonk, NY, USA). The categorical variables are presented as frequencies (percentages) and the continuous variables are presented as means ± standard deviations(SD). For the univariate analysis, the frequencies of the parameters were compared by Chi-square tests for categorical variables. Student’s *t*-tests were used to compare the continuous variables. The associations of the ICH scores with the 30-day mortality rate of the patients with basal ganglia haemorrhages were assessed with a binary logistic regression. P values less than 0.05 were considered statistically significant.

## Results

A total of 310 patients with putaminal haemorrhages were enrolled in our study (Table 1). There were 129 patients in the surgical treatment group and 181 patients in the optimal conservative treatment group. The haematoma volume in the surgical group was significantly larger than that in the conservative group (38.33 ± 18.35 VS. 24.78 ± 16.69; p < 0.001). The ICH score in the surgical group was significantly higher than that in the conservative group (1.71 ± 1.26 VS. 1.20 ± 1.42; p = 0.001). The 30-day mortality rate was not significantly different between the surgical and conservative groups (13.95% VS. 17.13%; p = 0.45). Only 3 patients were aged greater than or equal to 80 years, and 157 patients (50.6%) had an initial haematoma volume ≥30 ml.

**Table 1:** Baseline clinical characteristics of 310 patients with basal ganglia hemorrhage

	**Surgical group**	**Conservative group**	**p-value**
	**(N = 129)**	**(N = 181)**	
Age(mean ± SD)	56.59 ± 12.25	58.48 ± 12.78	0.192
Male/female	93/36	126/55	0.637
Hematoma volume(mean ± SD)	38.33 ± 18.35	24.78 ± 16.69	<0.001
Presense of IVH(n,%)	64(35.36%)	42(32.26%)	0.608
ICH score(mean ± SD)	1.71 ± 1.26	1.20 ± 1.42	0.001
Mortality rate of 30-day	18(13.95%)	31(17.13%)	0.45

**Figure 3:**
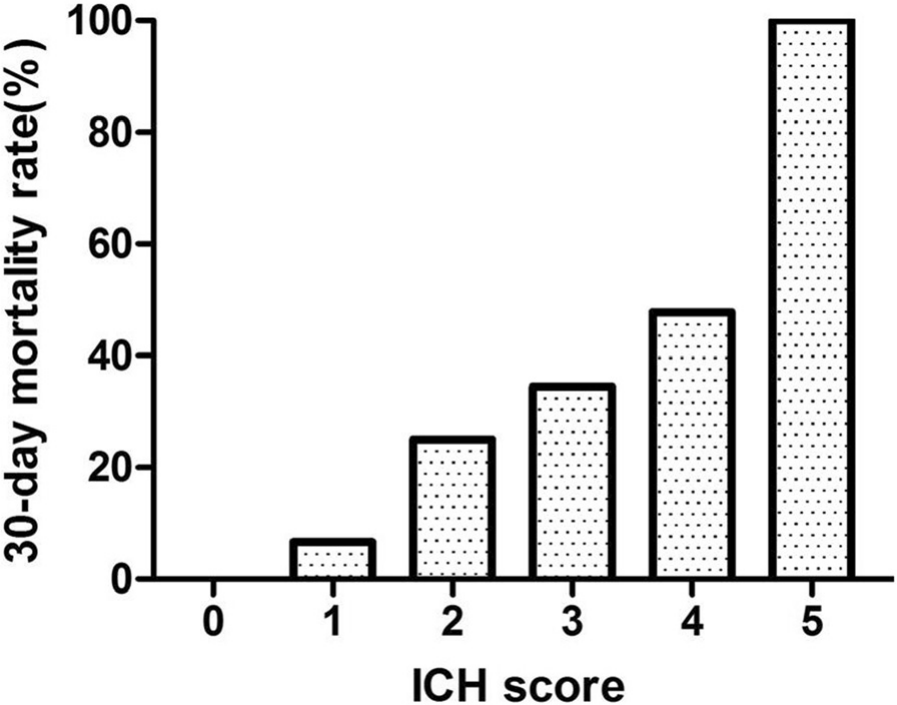
**The ICH score and 30-day mortality rate.** The each increase in the ICH score was associated with an increase in 30-day mortality rate.

There were 157 patients with basal ganglia haematomas volume ≥ 30 ml (Table 2). The 30-day mortality rate of the patients with haematoma volumes of ≥ 30 ml was significantly lower in the surgical group compared with those in the conservative group (15.6% VS. 62.3%; p = 0.002). The 30-day mortality rate of the patients with different ICH scores was different in the surgical group compared with that in the conservative group (Table 3). The 30-day mortality rate of the patients with ICH scores of 1 was significantly decreased in the surgical group compared to that in the conservative group (1.92% VS. 21.40%; OR = 0.072; p = 0.028). The 30-day mortality rate of the patients with ICH scores of 2 was obviously decreased (15.4% VS. 33.3%; OR = 0.365; p = 0.248). However, the 30-day mortality rate of the patients with ICH scores of 3 in the surgical group was even higher than that in the conservative group (37.50% VS. 22.20%; OR = 2.103; p = 0.661). There were 18 patients with basal ganglia haematomas volume of ≥ 30 ml and signs of cerebral herniation. The total 30-day mortality rate was up to 88.9%. The 30-day mortality rate in the surgical group and conservative group was 83.3% and 100%, respectively. However, there was no significant difference between the two groups (p = 0.526).Each increase in the ICH score was associated with an increase in the 30-day mortality rate (Figure 3). All of the patients with ICH scores of 0 survived, whereas none of the patients with ICH scores of 5 survived. The 30-day mortality rate of patients with ICH scores of 1, 2, 3 and 4 were 6.6%, 25%, 34.5% and 48.7%, respectively. A linear regression showed that the ICH score was negatively correlated with the 30-day mortality of the patients with basal ganglia haemorrhages (r = -0.798, p < 0.001).

**Table 2:** Clinical characteristics of 157 patients with basal ganglia hemorrhage (hematoma volume ≥30 ml)

	**Surgical group**	**Conservative group**	**p-value**
	**N = 96**	**N = 61**	
Male/female	72/24	42/19	0.4
Age(mean ± SD)	56.53 ± 12.50	57.95 ± 13.58	0.504
ICH score(mean ± SD)	2.04 ± 1.21	2.67 ± 1.21	0.002
Hematoma volume(mean ± SD)	45.13 ± 15.99	43.30 ± 15.77	0.481
Presence of IVH(n,%)	33(34.3%)	38(62.3%)	0.001
Signs of hernia (n,%)	12 (12.5%)	6(9.8%)	0.610
30-day mortality rate(n,%)	15(15.6%)	23(62.3%)	0.002

**Table 3:** The 30-day mortality of 157 patients with basal ganglia hematoma (≥30 ml) based on ICH score between surgical and conservative group

**ICH score**	**Surgical group**	**Conservative group**	**OR**	**p-value**
	**(30-day mortality rate)**	**(30-day mortality rate)**		
1	1.92% (N = 51)	21.40% (N = 24)	0.072	0.028
2	15.40% (N = 18)	33.30% (N = 9)	0.365	0.248
3	37.50% (N = 11)	22.20% (N = 17)	2.103	0.661
4	33.30% (N = 13)	54.50% (N = 11)	0.417	0.204
5	100.00% (N = 3)	None		

N, number of the patients.

None, no patient with an ICH score of 5 in the conservative group.

## Discussion

Our study showed that the 30-day mortality rate of patients with moderate basal ganglia haematomas was significantly decreased in the surgical group than that in the conservative group, and these results were similar to those of the other studies [10-13]. This is the first article about combining ICH score and hematoma volume as the indication of surgical removal of hypertensive basal ganglia hematoma. Haematoma volume is a very important factor in the surgical indication of patients with ICHs. In our study, the total 30-day mortality rate of patients with basal ganglia haematomas of 30 ml or more and ICH scores of 1 was significantly lower in the surgical group than in the conservative group. For the patients with ICH scores equal to 2, the 30-day mortality rate was obviously decreased in the surgical group compared with that that in the conservative group. It demonstrated that haematoma clot evacuation could limit the brain oedema and local ischaemia.

The 30-day mortality rate of patients with basal ganglia haematomas volume of ≥ 30 ml and signs of cerebral herniation was up to 90% even the hematoma was evacuated surgically within 24 h after the ictus. In our study, 16 of 18 patients with signs of herniation died within 1 week. The possible explanation was that only removal of haematomas might be insufficient to relieve the increased intra-cranial hypertension. The intra-cranial hypertension would increase again to severe values in few hours because of swelling of brain. Furthermore, the patients with ICH scores equal to 3, the 30-day mortality rate was higher in the surgical group than in the conservative group. Therefore, Treatment should be individualised for patients with basal ganglia haemorrhages. The ICH score was highly associated with the 30-day mortality rate of patients with basal ganglia haemorrhages, which was similar to other studies [14,15]. Therefore, the ICH score would provide a standard assessment tool, which can be determined rapidly and easily, for treatments selection of patients with hypertensive basal ganglia haemorrhage.

This study demonstrated that surgical intervention would decrease the 30-day mortality rate of patients with hypertensive basal ganglia haematomas of ≥ 30 ml and ICH scores of 1 or 2. But this retrospective study had some limitations. The hematoma removal was through different surgical procedures. Some patients with large haematoma didn’t receive surgical intervention because of economy; However, a subset of patients with basal ganglia hematoma volume of < 30 ml and shift of midline ≥ 5 mm received surgical removal of hematoma. A more definitive conclusion will be achieved from the future trial.

## Conclusion

Patients with basal ganglia haematomas volume ≥ 30 ml and ICH scores of 1 or 2 could benefit from micro-surgical interventions. ICH scores should be assessed as part of the surgical indications and routine clinical care.

## Abbreviations

ICH score: Intracerebral hemorrhage score.

## Competing interests

I disclose that we haven’t received any reimbursements, fees, funding, or salary from an organization in the past five year that may in any way gain or lose financially from the publication of this manuscript, either now or in the future. We don’t hold any stocks or shares in an organization that may in any way gain or lose financially from the publication of this manuscript, either now or in the future. We don’t hold or aren’t applying for any patents relating to the content of the manuscript. We haven’t received any reimbursements, fees, funding, or salary from an organization that holds or has applied for patents relating to the content of the manuscript. We don’t have any other financial competing interests. There aren’t any non-financial competing interests (political, personal, religious, ideological, academic, intellectual, commercial or any other) to declare in relation to this manuscript.

## Authors’ contributions

Author contributions to the study and manuscript preparation include the following. Conception and design: all authors. Acquisition of data: HLi and YZ. Analysis and interpretation of data: HLiu and HLi. Drafting the article: HLiu. Revising the article: JL and XW. Reviewed the submitted version of manuscript: all authors. Approved the final version of manuscript on behalf of all authors: CY. Study supervision: CY.

## Pre-publication history

The pre-publication history for this paper can be accessed here:

http://www.biomedcentral.com/1471-2377/14/141/prepub
